# Growth inhibition of an *Araucaria angustifolia* (Coniferopsida) fungal seed pathogen, *Neofusicoccum parvum*, by soil streptomycetes

**DOI:** 10.1186/1471-2180-13-168

**Published:** 2013-07-18

**Authors:** Fernando Rostirolla Dalmas, Leandro Astarita, Luigi DeFilippis, Elisabeth Magel, Hans-Peter Fiedler, Robert Bauer, Rüdiger Hampp

**Affiliations:** 1 Laboratory of Plant Biotechnology, Bioscience Institute, Pontifícia Universidade Católica do Rio Grande do Sul, Ipiranga Avenue, 6681, Building 12A, CEP: 90619-900, Porto Alegre, RS, Brazil; 2 Centre for Environmental Sustainability, Faculty of Science, University of Technology Sydney, P.O. Box 123, Broadway/Sydney, NSW 2007, Australia; 3 Wood Biology, Department of Wood Science, University of Hamburg, Leuschnerstrasse 91d, D-21031, Hamburg, Germany; 4 Microbiology/Biotechnology, University of Tübingen, Auf der Morgenstelle 28, D-72076, Tuebingen, Germany; 5 Plant Systematics, University of Tübingen, Auf der Morgenstelle 1, D-72076, Tuebingen, Germany; 6 Physiological Ecology of Plants, University of Tübingen, Auf der Morgenstelle 1, D-72076, Tuebingen, Germany

**Keywords:** Antibiosis, Actinomycetes, Brazil pine, Secondary compounds, Anti-fungal compounds, *Neofusicoccum*

## Abstract

**Background:**

Araucariaceae are important forest trees of the southern hemisphere. Life expectancy of their seedlings can largely be reduced by fungal infections. In this study we have isolated and characterized such a fungus and investigated the potential of *Streptomyces* Actinobacteria from the respective rhizosphere to act as antagonists.

**Results:**

The pathogenic fungus from *Araucaria angustifolia* seeds was identified by morphological markers (pore-associated Woronin-bodies) as belonging to the Pezizomycotina. Molecular data identified the fungus as *Neofusicoccum parvum* (Botryosphaeriaceae). Co-cultures on agar of this fungus with certain streptomycete isolates from the rhizosphere, and from the surface of *Araucaria* roots significantly reduced the growth of the fungus. HPLC analysis of the agar yielded streptomycete-specific exudate compounds which were partly identified. There were differences in compounds between single (bacteria, fungus) and dual cultures (bacteria + fungus).

**Conclusion:**

Streptomycetes from the rhizosphere of Araucariaceae produce exudates which can suppress the development of pathogenic fungi in their seeds.

## Background

Tropical and subtropical forests once covered large areas of Central- and South America. Due to high rates of deforestation up to the 80ies of the last century, and also wildfires, large areas are now grasslands or campos [1], or are used for agricultural purposes (own observations). Species of the coniferous genus *Araucaria* are important members of tropical and subtropical forests of the southern hemisphere [2]. Among them, Brazil pine (*Araucaria angustifolia* [Bertol.] Kuntze) was one of the most important species, economically and ecologically [3,4], occurring in mountain areas (above 800 m) of Southern Brazil, and dominated the forest vegetation [3]. Due to severe clear cutting and fires, native *Araucaria* forests today occupy only 1% of the original area occupied [4,5]. Brazil pine is thus an endangered species [6]. Recent investigations, however, show that under undisturbed conditions forest land starts to invade the grasslands again [7]. Araucariaceae represent very ancient gymnosperms and are also called “living fossils”. According to largely missing literature on this subject, these trees are obviously not very sensitive to fungal pathogens in comparison to conifers of the northern hemisphere. In the latter, root-rot inducing species such as *Heterobasidion* spec. cause considerable losses in wood production [8,9]. There is, however, a recent report on root and crown rot in *A. angustifolia*, caused by *Phytophthora cinnamomi*[10], and most recently, Dalmas and Astarita (unpublished observation) detected a fungal pathogen in *A. angustifolia* seedlings, which severely inhibited seedling development.

With regard to biocontrol, streptomycetes, which are an important part of bacterial communities of the rhizosphere, have attracted special attention. Streptomycetes produce and release a wide variety of secondary metabolites. Approximately 7,600 out of 43,000 biologically active secondary metabolites, such as antibiotics, have been characterized from streptomycetes [11]. When released to the soil, these may contribute to biocontrol, including the induction of systemic resistance in streptomycetes-colonised plants [12-14]. In studies with spruce seedlings, it could be shown that streptomycetes from the rhizosphere of a spruce stand could systemically improve resistance of seedlings against fungal infection [15].

It was the aim of this study to identify the newly isolated fungal pathogen of *A. angustifolia* seeds and screen for rhizosphere streptomycetes which, upon germination on ground, can affect the growth of this pathogen. Furthermore, we present a list of exudate compounds produced by the fungus-inhibiting bacteria in single culture, and alterations due to the co-culture with the fungal pathogen.

## Results and discussion

### The pathogenic fungus on *A. angustifolia* seedlings: effects and identification

**Figure 1:**
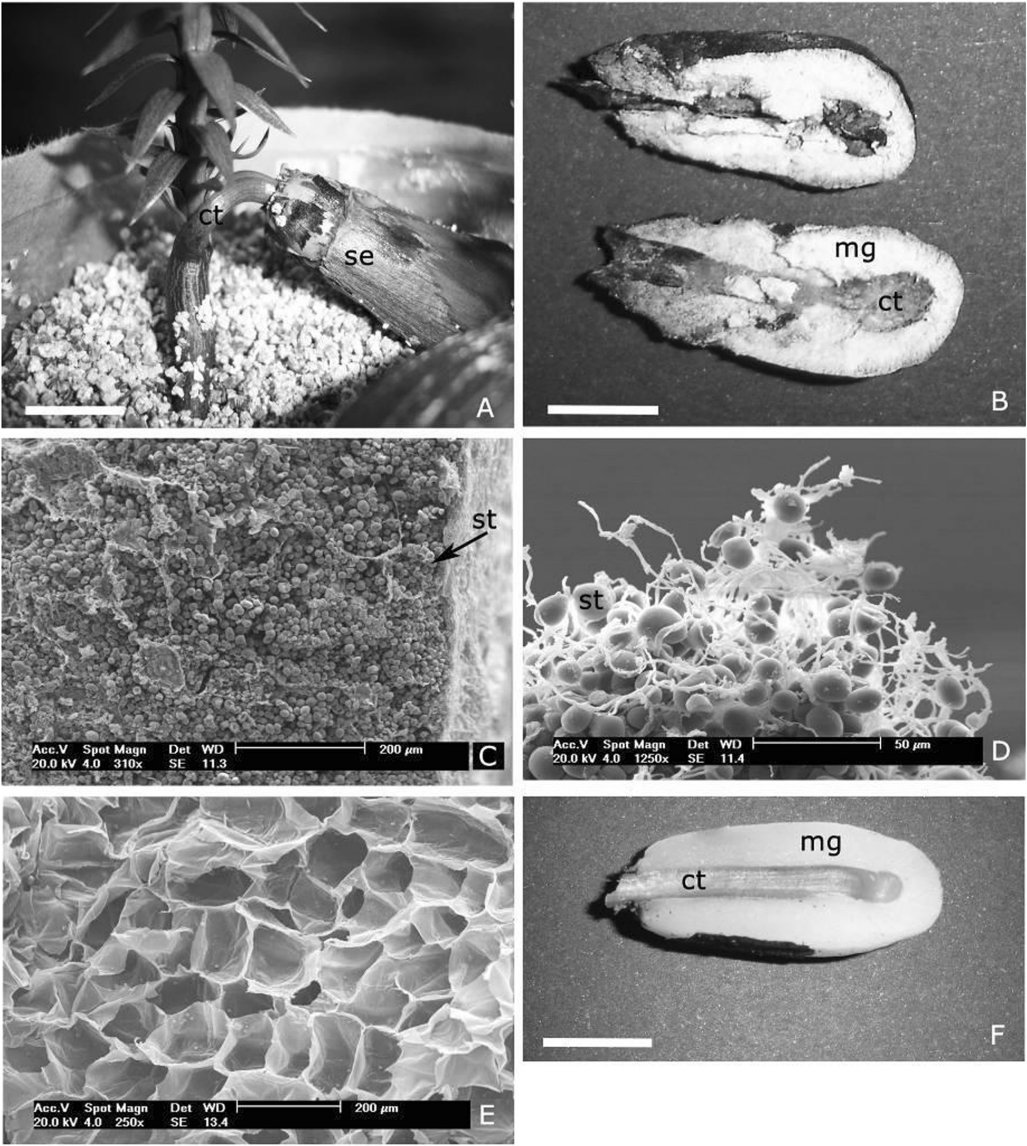
***Neofusicoccum parvum *infection of *A. angustifolia *seedlings (Bar = 1 cm in A, B, F). A**, Seedling; **B**, Megagametophyte and cotyledons infected with the fungus; **C**, Scanning electron microscopy of infected megagametophyte tissue that surrounds the cotyledon; **D**, Starch grains covered by hyphae; **E**-**F**, Non infected tissues. All images were taken from plants/tissues after 50 days of germination. ct - haustorial cotyledon, se - seed, mg – megagametophyte, st - starch grain.

After 50 days of germination, about 30% of *Araucaria* seedlings were infected by a fungus that promoted the death of the cotyledons and interrupted the connection between the seedling and the megagametophyte (Figure 1A, B). Of these, about 50% died, and the surviving ones showed delay in plant development. After 150 days, 52.3% of surviving plants with retarded development were dead. The cause for delayed development or seedling death might be attributed to the early interruption in the carbon and nutrients transfer from the megagametophyte to the embryonic tissues. Electron microscopy analyses showed the presence of high amounts of starch grains in the megagametophyte of infected seedlings (Figure 1C, D), compared with the non-infected tissue (Figure 1E, F).

The natural infection of the *A. angustifolia* seeds by the fungus might have happened during cone maturation and before seed dispersion. The fungus infected specifically the megagametophyte tissue and promoted necrosis of the seed-enclosed region, and the cotyledons, after their emergence. The first visible symptoms were the decay of the cotyledons and seed browning. In this species, the cotyledons act as a haustorial organ by transferring the reserves from the megagametophyte to the embryonic axis [16], supporting the seedling growth until about 70 to 120 days [17,18]. The early cotyledon interruption leading to seedling death or delayed plant development, significantly reduced the chances for seedling establishment.

ITS sequencing of the fungal isolate with the primer pairs ITS1 and ITS4 ([19], accession number ITS [JN811822]) yielded the highest homologies (100%) with *Neofusicoccum parvum*/*N. ribis* and *Botryosphaeria parva*, all members of the Botryosphaeriaceae. This is due to the fact that *Neofusicoccum parvum* is the anamorph of *Botryosphaeria parva*[20]. *N. parvum* and *N. ribis* were originally considered to be part of the *Botryosphaeria dothidea* complex [21]. Currently, these two *Neofusicoccum* species, together with three cryptic species isolated from *Syzygium cordatum* in South Africa, are regarded as forming a unique group, named the *N. parvum/N. ribis* complex [22]. However, only *Neofusicoccum parvum* has been frequently associated with brown streaking and necrosis of wood [23,24]. Based on genomic markers, Pavlic et al. [22] identified five groups*, N. parvum, N. ribis*, and three distinct lineages within the *Np/Nr* complex. Sequences of ITS [JN811822], EF-1a [JN811823], BT [JN811824]*, BotF15* [JN811825], or RPB2 [JN811826] of the unknown fungi, did not contain one of the SNPs characteristic for *N. ribis* or the members of the three lineages *N*. sp R1, *N*. sp R2, or *N*. sp R3. Alignment of the ITS-sequences revealed one indel at position 118 to *N. ribis* (missing G) and one SNP at position 379 to *N. parvum* (T). Based on these data and a report about the identification of *N. parvum* on *A. heterophylla*[25] we suggest this fungus is *N. parvum*. This fungus has been reported in both Brazil and Australia.

**Figure 2:**
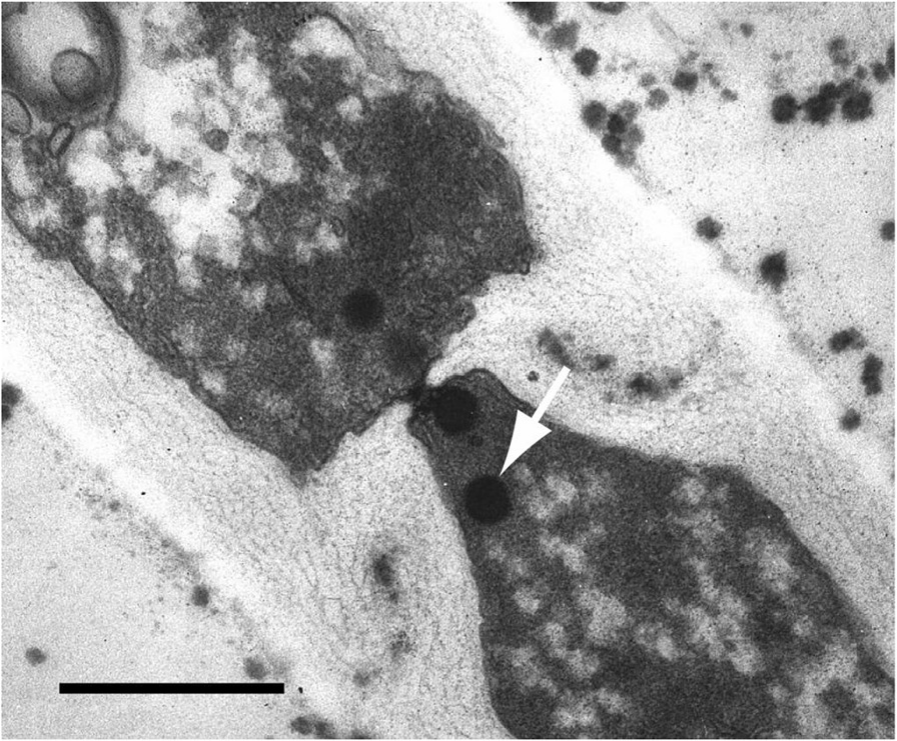
**Section through a septum of *Neofusicoccum parvum *showing a simple pore associated with Woronin-bodies (one is indicated by an arrow).** Scale bar = 0.5 μm.

Electron microscopy of fungal hyphae strongly supports the sequence data. Figure 2 shows septa with simple pores having more or less rounded lips. The pores are associated with Woronin-bodies which identifies the fungus as ascomycete, belonging to the subphylum Pezizomycotina [26,27].

Fungi of Botryosphaeriaceae occur in a wide diversity of plants and can act in different ways, as primary or opportunistic pathogens, but also as endophytes or saprobes [28,29]. Since such fungi have also been reported to affect Araucariaceae, such as the recently discovered *Wollemia nobilis* in Australia, as well as *Araucaria* spec. in New Zealand [30], biocontrol properties of Australian streptomycetes are not only of local interest.

### Rhizosphere streptomycetes with biocontrol potential and their exudates

**Figure 3:**
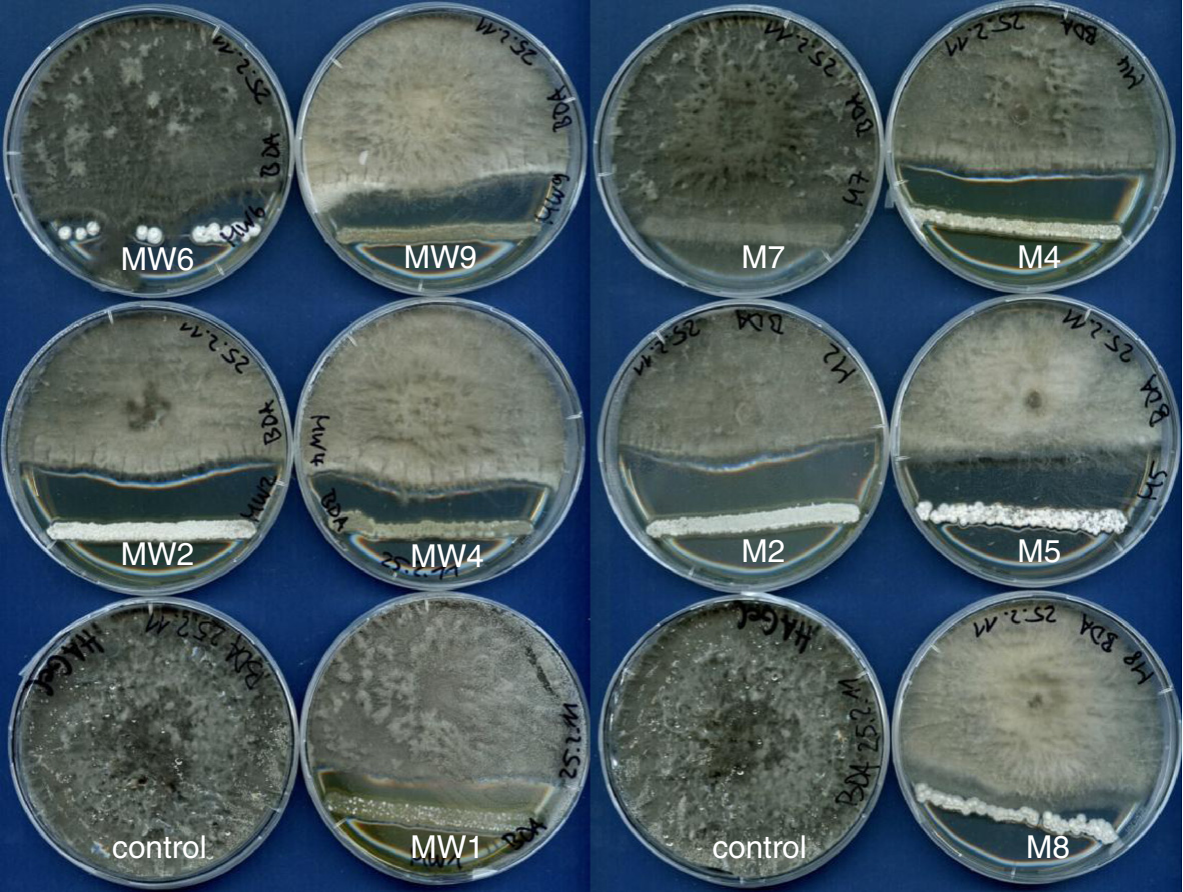
**Co-cultures of streptomycete isolates with the plant pathogenic fungus *Neofusicoccum parvum.*** The fungal isolate is located in the center of the Petri dish. Mxy identifies the different Streptomycete isolates. M, isolates from rhizosphere soil; MW, isolates from the surface of *Araucaria* roots.

We thus screened streptomycete isolates from Australian *Araucaria* stands for potential inhibitors of fungal growth. As bacterial populations differ between bulk soil and root surface, we tried to isolate bacteria from both sources (“W” stands for root surface). Co-culture experiments showed different degrees of growth inhibition (Figure 3). Most effective isolates were M2, M4, M5, MW2, MW4 and MW9. Sequence analysis of 16S rDNA demonstrated that these isolates were streptomycetes. 16S ribosomal RNA gene homologies (above 97%) were with *Streptomyces albulus* (JX235956; M8), *Streptomyces chattanoogensis* (KC292488; M5, MW6) and *Streptomyces* sp. Ac189 (JQ780468; MW2 , M4, M7, MW1, MW9, M2) or *Streptomyces celluloflavus* (NR041150/AB184476; MW2, M4, M7, MW1, MW9, M2).

**Figure 4:**
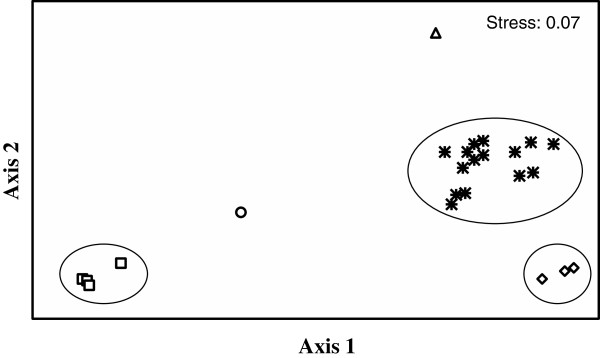
**Association statistics of the 24 exudate compounds, listed in Table **1**.** This approach illustrates that the inhibition of the fungus in co-culture was dependent on the presence of compounds of group 1 (component 1–4; □) and group 2 (component 16–18; ◊). For numbers of the relevant compounds see Table 1: □ 1,2,3,4; ◊ 16–18; ○ 22; Δ 13; ӿ 5–12, 14–15, 19–21, 23–24.

From single cultures of bacterial isolates and fungus/bacteria co-cultures on agar, 24 different compounds could be identified by comparing the HPLC-MS profiles of the respective agar extracts with an in-house HPLC-UV–VIS database (Table 1). The mix of the different exudates was to some degree isolate-specific. Multi dimensional statistical (MDS) data analysis illustrates which individual cultures and co-cultures form clusters, and which cultures could be considered similar to one another, on the basis of patterns and combinations due to the presence or absence of exudate compounds. This approach indicates that the inhibition of the fungus in co-culture (Figure 3; MW2, 4, 9; M2, 4, 5) was dependent on the presence of compounds of two groups (Figure 4; Table 2). These are group 1, made up by compounds 1, 2, 3 and sometimes 4 (Figure 4; □), and group 2, consisting of compounds 16, 17, and 18 (Figure 4; ◊), each enclosed by circles. Group 1 consists of a ß-carboline alkaloid usually extracted from Actinomycetes (1-acetyl-β-carboline, 1 in Table 1), containing an indole tricyclic ring and is cytotoxic, anti-microbial and an enzyme inhibitor [31]. The other three metabolites in this group are polyene macrolide antibiotics, containing a lactose ring and act against ergosterol of fungal membranes. Filipin is more toxic than lagosin and all three cause excess leakage of K [32]. Group 2 consist of a peptide antibiotic (stenothricin, 16) that affects glycolytic and lipolytic proteins, and inhibits cell wall formation [33]. The other two compounds (17, 18) are auxins or auxin antagonists (plant hormone derivatives) and may affect many aspects of plant growth and development [34]. Compounds 17 and 18 were generally not released or present from single cultures of either bacteria or fungus, and this is consistent with their roles more directly in plants. Two other well separated metabolites are worth mentioning (i.e. Figure 4/Table 1, 13 and 24). Thiolutin (Δ) is a well studied broad spectrum indole alkaloid which inhibits energy metabolism, RNA synthesis (RNA polymerase), glucose metabolism and carbon use [35]. N-hydroxy phenyl acetic acid methyl ester is a derivative of indole propionic acid and is a weak alkaloid and anti-microbial compound, acting mainly against Gram-negative bacteria [34]. Most effective in the inhibition of fungal growth are combinations and the presence of compounds belonging to both group 1 and group 2, however, not all metabolites included in these groups are apparently necessary for inhibition.

**Table 1:** **Compilation of compounds identified by HPLC-MS from exudates released into the agar by the different streptomycte isolates, singly or in co-culture with *N. parvum***

**Number**	**Compound**	**Number**	**Compound**
1	1-Acetyl-β-carboline	13	Thiolutin
2	Lagosin	14	NL 19 KF RT 3.59
3	Filipin	15	Tu 6121-H
4	Pentamycin	16	Stenothricin
5	Desferrioxamine B	17	N-Acetyl-tryptophan
6	Aerobactin	18	Indole-3-acetic acid
7	Phenyl acetic acid amide	19	Indole-3-proprionic acid
8	N-Acetyl-tyramin	20	Tryptophol
9	Pyridin-2,3-dicarboxylic acid	21	Simocyclinone C4
10	Platomycin B	22	HAG 010767-A2
11	L-tryptophan	23	4-Dihydroxy-benzyl propionic acid
12	Ketomycin	24	3-Hydroxy phenyl acetic acid methyl ester

**Table 2:** **Substances released into the agar by the different isolates singly, or in co-culture with *N. parvum***

**Origin of isolate/co-culture**	**Streptomycete isolates**	**Identified metabolites**
Rhizosphere	M2	1,2,3,4,5,6,7.13
	M4	1,2,3,4,7,13
	M5	1,2,3,4,8,9,10
	M7	8,14,15
	M8	6,8,11,15
Root surface	MW1	5,12
	MW2	1,2,3,4,12
	MW4	1,2,3,4,13
	MW6	1,7
	MW9	1,2,3,4,7,12,13
Rhizosphere bacteria + *N. parvum*	BM2	1,2,3,16,17,21,23,24
	BM4	1,2,3,16,17,18
	BM5	1,2,3,4,17,18,19,22
	BM7	14,15,17,18
	BM8	15,16,21
Root surface bacteria + *N. parvum*	BMW1	1,2,3,5,21
	BMW2	1,2,3,4,13,16,17,18,23,24
	BMW4	1,2,3,4,16,17,18,19,20,21
	BMW6	13,21,30,31,32
	BMW9	1,2,3,7,16,17,22

In co-culture, substances can result from both organisms. M, isolates from rhizosphere soil; MW, isolates from the surface of *Araucaria* roots.

We could not test the effects of single compounds or combinations thereof, as they are not commercially available. They only can be obtained from preparative batch cultures. We have done this before [36], but due to the considerable necessary efforts, this could not be done for the present investigation.

**Figure 5:**
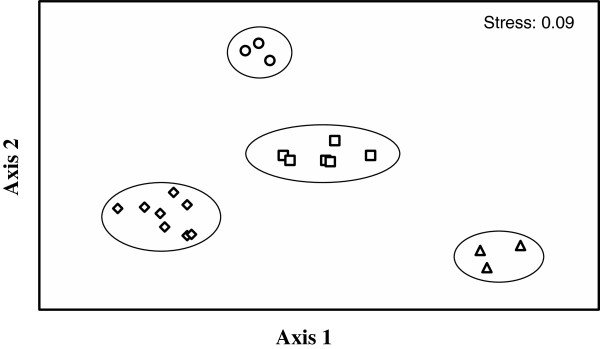
**Association statistics of the streptomycete isolates or their co-cultures with *N. parvum *and the respective exudates.** Fungus-inhibiting bacteria together with their exudates (singly or in combination with the fungus; □, ○, Δ) separate well from those causing little or no inhibition (◊). □ M2, 4, 5; MW 2, 4, 9; ○ BM2, 4, 5; Δ BMW2, 4, 9; ◊ M7, 8; MW1, 6; BM7, 8; BMW1, 6. M, isolates from rhizosphere soil; MW, isolates from the surface of *Araucaria* roots. B, co-cultures with the Brazilian fungus (*N. parvum*).

Association statistics of the streptomycete isolates and their inhibitory effects on *N. parvum* revealed that under co-culture, the strong inhibitory BM (BM2, 4, 5; Figure 5 ○) and BMW groups (BMW2, 4, 9; Figure 5 Δ, encirceld) were even more widely separated. This indicates that the co-cultures showing the highest degree of inhibition were not only different from one another but also very different from the rest of the non-inhibiting cultures with regard to their exudates profiles.

**Figure 6:**
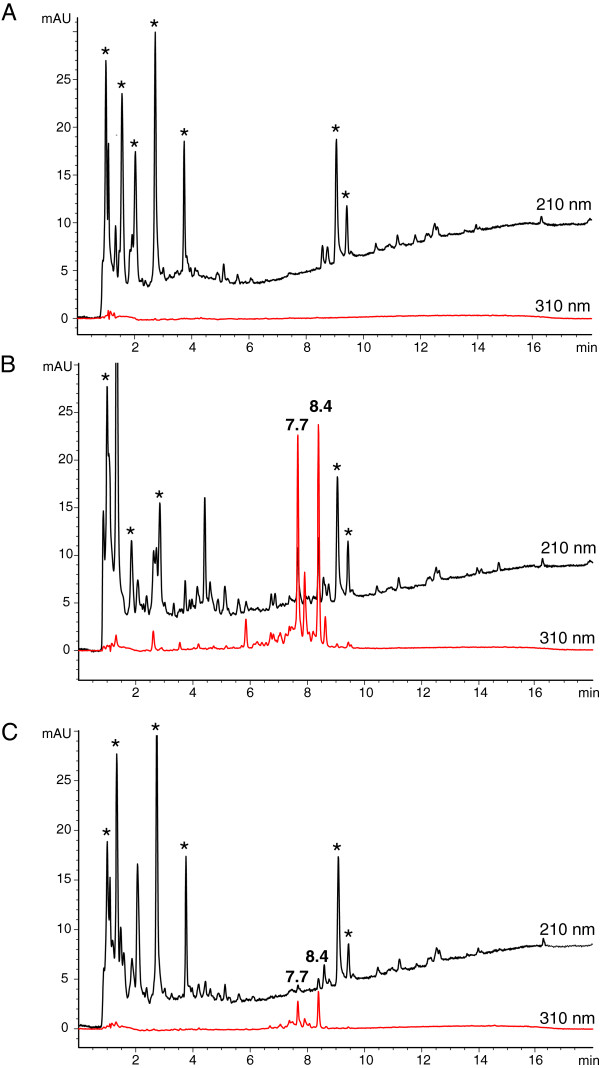
**HPLC analysis of agar extracts obtained from single and dual cultures in Petri dishes.** The eluate was monitored at 210 and 310 nm. **A)***Neofusicoccum parvum*, **B)** bacterial isolate M5, **C)** co-culture of bacterium and fungus. Peaks labelled with retention times of 7.7 and 8.4 min represent tetraene-polyene macrolides of the nystatin-type, those with an asterix indicate agar constituents.

Exudates released from the Streptomyces isolate M5 and *N. parvum* in single culture and after co-culture were characterized by HPLC in more detail (Figure 6). Interestingly, the two identified bacterial peaks (tetraene-polyene macrolides; 7.7 and 8.4, Figure 6B and C) had decreased in amounts in the presence of the fungus. As detailed before, the macrolide antibiotics are active against yeasts, molds and filamentous fungi, and can cause membrane distortions and leakage of K [37]. The decline in amounts indicates that the fungus also responds to the *Streptomyces,* possibly by taking up these antibiotics which then affect fungal metabolism. On the other hand, the fungus does not release many compounds into the agar, at least not such ones with low polarity which can be identified by reverse phase HPLC.

In recent studies we could show that certain streptomycete isolates can completely abolish disease development caused by the infection of spruce seedlings with the root pathogenic fungi *Armillaria* spec., and *Heterobasidion* spec. [38,39]. This effect could be attributed to an antibiotic, isolated from the streptomycete [36]. The present study confirms the biocontrol function of many soil bacteria, and especially of streptomycetes. It also shows that combinations of exudates are obviously more relevant than the application of single compounds. Although the investigation of effector combinations is only a very little step towards the understanding of microbe interactions in the complex rhizosphere. In ongoing experiments we will try to find out whether the co-culture effects can be simulated by the addition of these compounds (as far as available), and whether the infection of *Araucaria* seedlings by the fungus can be prevented by co-culture with the respective streoptomycete isolates. In addition, we have started to screen a range of streptomcete isolates obtained from Brazilian *Araucaria angustifolia* stands for their biocontrol function. For application, spores of efficient bacteria could then be added to *A. angustifolia* seeds to counteract *N. parvum* infection.

## Conclusions

Streptomycetes from the rhizosphere of Araucariaceae produce exudates which can suppress the growth of pathogenic fungi in their seeds. The focus of this contribution is on the effect of bacteria from Australian sources on a Brazilian tree species (*A. angustifolia*). However, our most recent studies show that the potential biocontrol properties of Brazilian rhizosphere bacteria are very similar to those of Australian isolates. Thus, the bacterial impact is not restricted to the respective source of bacteria, or bacteria/species of Araucariaceae.

## Methods

### Culture of *Araucaria angustifolia* seedlings

Mature cones of a single *Araucaria* specimen were collected in the *Araucaria* forests of the Pró-Mata Centre for Research and Conservation of Nature (29°29′28.8”S, 50°11′9.8”W), São Francisco de Paula, Rio Grande do Sul, Brazil, in April 2009. The cones were disassembled into single seeds, which were disinfected with sodium hypochlorite (2% active chlorine) for 20 min, followed by 0.3% Benlate fungicide (Dupont, Belle, WV, US) for 10 min, and rinsed with sterile distilled water. The seeds were then placed in polyethylene bags and maintained at 0°C until use. Seeds were placed on sterile filter paper embedded in 10 ml of sterile distilled water in Petri dishes, and allowed to germinate. After the start of germination (day 0), seedlings were transferred to polyethylene jars (1.9 l) containing moist sterile vermiculite. The jars were kept wet by the addition of 100 ml of sterile distilled water at 10-day intervals. All jars were kept at 25 ±2°C with light intensity of 31 μmol m^-2^ s^-1^ in a 16-h photoperiod. The natural occurrence of the pathogenic fungus and plant mortality were evaluated at days 50 and 150. The evaluation period was chosen according to the pattern of depletion of seed reserves. The plant growth is strongly dependent on carbohydrate import from seed until 70 – 80 days after germination [17] and the seed reserves are apparently exhausted approx. 100 days after planting [40].

### Isolation and culture of the fungal pathogen

Fungal infection was not observed on seeds before they had developed. The first disease symptoms consisted of cotyledon browning and abscission, followed by browning and hardening of the megagametophyte. The fungus was isolated from about 50-days-old seedlings. For this purpose the megagametophyte and the cotyledons were removed, superficially disinfected in 96% ethanol (1 min) and submersed in 1% sodium hypochlorite for 10 minutes. The material was desiccated in a laminar flow bench and the megagametophyte was separated from the cotyledons. Infected tissues were transferred to tubes with PDA medium (potato dextrose agar, Acumedia Manufactures, Inc. Lansing, MI, USA) using a sterile platinum loop. Tubes were incubated at 26°C for 7 days and examined for fungal growth. The emerged fungus was transferred to fresh PDA medium. Continuous culture was on ISP-2 agar [41].

The microorganism was found in all plants showing symptoms of infection. The pathogenicity test was performed by using healthy seeds excised from mature cones collected in 2011. Seeds were disinfected as previously described and scarified by removing the integuments from the seed tip [40], exposing the megagametophyte. Scarified seeds were incubated at 25°C in darkness with the fungus. For this purpose, seeds were placed in a tray and partially covered with sterile water containing mycelium. Mycelial plugs (1.5 cm diameter) of 14-day-old cultures of the isolate were homogenized in 10 ml sterile water. Controls consisted of sterile water, supplemented with an agar plug without fungus. Trays were maintained on an orbital shaker (50 rpm) for 48 h. After this period, seeds were added, and the resulting seedlings were transferred to polyethylene jars, as described above. Each experiment consisted of two replicates with 33 seeds each. When seeds were incubated in the presence of the fungus, 42% of germinated plants developed the disease and died up to 70 days after inoculation, presenting the same symptoms previously observed.

### Isolation and culture of bacteria

Because bacteria from bulk soil can be different from those attached to the root surface, they were extracted from both roots and sandy soil under *Araucaria cunnighamii* trees. The location was Wild Cattle Creek State Forest, Megan NSW, Australia (30°16′40”S, 152°50′15”E). Soil samples were taken in February from the respective “rhizosphere”, which was defined as the root containing organic layer after removal of the uppermost undigested litter layer. Rhizosphere sampling was between 3 to 8 cm from the surface and at a distance of approximate 2 m from the tree trunk. Three randomly taken samples were mixed and dried at 60°C. About 500 mg of dried soil were extracted with sterile 50 ml HNC medium, selecting specifically for Actinomycetes (yeast extract, 60 g; sodium dodecyl sulfate, SDS, 0.5 g; CaCl_2_, 0.5 g dissolved in 1 l de-ionized water [42,43]). The medium contained glass beads, and the samples were kept on a rotatory shaker at 200 rpm and 42°C. The resulting suspension was filtered through cotton. Filtrates were diluted 10 or 100 fold with water, and 50 μl plated on Petri dishes with ISP-2 agar [41] (yeast extract, 4 g; malt extract, 10 g; glucose, 4 g; agar (Serva, Germany), 20 g dissolved in 1 l tap water). After autoclaving the following antibiotics were added (per l): 50 mg cycloheximide (in 10 ml methanol), 50 mg nystatin (in 10 ml methanol) and 100 mg nalidixinic acid (in 10 ml H_2_O; pH 11). The dishes (5 to 10 parallels) were sealed with Parafilm and incubated at 27°C. When single colonies appeared, they were transferred to new plates. When the cultures were pure, they were kept on ISP-2 agar, containing additionally CaCl_2_ (malt extract, 10 g; yeast extract, 4 g; glucose, 4 g; CaCl_2_* 2 H_2_O, 1.47 g; agar agar, 20 g; dissolved in 1 l de-ionized water; pH 7).

### Co-culture of bacteria and fungi

For testing the effect of bacteria on fungal growth, dual cultures were used. The fungal inoculum was excised from the actively growing edge of a fungal colony using the wide end of a Pasteur pipette and transferred to the center of an ISP-2 [41] agar in a 9-cm-diameter Petri dish. Bacterial isolates were taken from a suspension culture in HNC medium at an OD_650_ of about 0.6, and applied to the edge of the Petri as a thin line of about 4 cm in length. The distance between both inocula was at least 3.5 cm, and both were physically separated by the medium. The Petri dishes were incubated for 2 weeks at 20°C in darkness (at least 2 independent trials with 4 parallels each). Because of the fast fungal growth, bacteria were added 1 week earlier to the Petri dish.

### Taxonomic characterization of the pathogenic fungus and of the bacterial isolates

For molecular characterization, DNA was prepared using the GenElute bacterial genomic DNA kit from Sigma (Taufkirchen, Germany) according to the manufacturer´s instructions. Genomic DNA was used as template for PCR-amplification of the rDNA-ITS region, a portion of gene encoding translation elongation factor 1 alfa (EF-1a), the Bt2 region of the ß-tubulin gene, a portion of RNA polymerase II subunit (RPB2), and locus *BotF15*, an unknown locus containing microsatellite repeats [22]. The respective primers are given in Table 3. The PCR was carried out with the Taq PCR Core Kit (Qiagen, Hilden, Germany). PCR products were purified using a QIAquickPCR Purification Kit (Qiagen, Hilden, Germany). Sequencing was done commercially (MWG-Biotech, Ebersberg, Germany).

**Table 3:** Compilation of primers used for the amplification of ITS, EF-1a, ß-tubulin, RPB2, BotF15 of the fungus, and of partial 16S rDNA region of the bacteria

	**Forward primer 5′ – 3′**	**Reverse primer 5′ – 3′**	**Literature**
ITS	ITS1: TCCGTAGGTGAACCTGCGG	ITS4: TCCTCCGCTTATTGATATGC	White et al. 1990 [15]
EF-1a	EF-AF: CATCGAGAAGTTCGAGAAGG	EF-BR: CRAT GGT GAT ACC RCG CTC	Pavlic et al. 2009 [18]
ß-tubulin	Bt2a: GGTAACCAAATCGGTGCTGCTTTC	Bt2b: ACCCTCAGTGTAGTGACCCTTGGC	Pavlic et al. 2009 [18]
RPB2	RPB2bot6F: GGTAGCGACGTCACTCCC	RPB2bot7R: GGATGGATCTCGCAATGCG	Pavlic et al. 2009 [18]
*BotF15*	Bot15: CTGACTTGTGACGCCGGCTC	Bot16: CAACCTGCTCAGCAAGCGAC	Pavlic et al. 2009 [18]
16S rDNA	27 F: AGAGTTTGATGCTCAG	765R: CTGTTTGCTCCCCACGGTTTC	Coombs and Franco 2003 [33]

### Secondary metabolites produced by the bacterial isolates and co-cultures

Bacterial isolates were applied to the Petri dish as thin lines with a distance of about 3.5 cm in between. For co-cultures, the fungus was added to the same plate but one week later. After culturing for 10 days, the intermittent agar stripes were cut out, wrapped with Parafilm (both ends open) and frozen at −20°C. For the analysis of released secondary metabolites, the frozen stripes were thawed between two fingers and the resulting liquid squeezed into Eppendorf vials. The samples were dried under vacuum centrifugation (Speedvac, Savant Instruments, Holbrook, NY, USA) and the residues dissolved in 100 μl methanol. Methanol has enough solubility properties to dissolve both, less lipophilic and lipophilic compounds out of a dry highly concentrated sample. A further advantage of methanol-dissolved samples is their compatibility with reversed-phase HPLC using water as starting solvent in gradient elution. When co-cultures were investigated, the clear agar (visibly free of both micro-organisms) between bacterium and fungus was used.

In order to understand patterns of variation in antibiotic compounds within and amongst cultures and co-cultures, PRIMER versions 5.2.7 and 6.0 [44] were used. This software converts a set of variables into a few dimensions so that individual variations are condensed into a set of two axes (i.e. multi dimensional scaling, MDS). Such graphical analysis helped to identify exudate compounds and cultures which tended to cluster together and have high similarities. The cluster procedure was an average linking one, and all similarities used were based on Eucledian distances. Exudate compounds identified were scored ‘1’ for the presence, and ‘0’ for the absence of the compound.

### HPLC analysis of streptomycete secondary metabolites

The chromatographic system consisted of a HP 1090 M liquid chromatograph equipped with a diode-array detector and HP Kayak XM 600 ChemStation (Agilent Technologies, Waldbronn, Germany). Multiple wavelength monitoring was performed at 210, 230, 260, 280, 310, 360, 435 and 500 nm, and UV-visible spectra measured from 200 to 600 nm. Five-μl aliquots of the samples were injected onto a HPLC column (125×3 mm, guard column 20×3 mm) filled with 5-μm Nucleosil-100 C-18 (Maisch, Ammerbuch, Germany). The samples were analyzed by linear gradient elution using 0.1% *ortho*-phosphoric acid as solvent A and acetonitrile as solvent B, at a flow rate of 0.85 ml min^-1^. The gradient was from 4.5% to 100% for solvent B in 15 min with a 3-min hold at 100% for solvent B. Evaluation was carried out by means of an in-house HPLC-UV–vis database which contains nearly 1000 reference compounds, mostly antibiotics [45].

### Electron microscopy

The megagametophyte tissues were evaluated on those *A. angustifolia* seedlings, which showed interrupted cotyledon connections. Samples were fixed in 0.05 M sodium phosphate buffer (pH 8.0) containing 2% glutaraldehyde. The samples were gradually dehydrated in acetone, critical-point dried, sputter-coated with gold and observed by scanning electron microscopy.

## Abbreviations

HPLC: High-performance liquid chromatography; ITS: Internal transcribed spacer; PCR: Polymerase chain reaction; UV–VIS: Ultraviolet–visible.

## Competing interests

The authors declare to have no competing interests.

## Authors’ contributions

RH initiated the investigation, and together with LDF, EM acquired the soil samples. In co-operation with LDF and EM, RH prepared the manuscript. The fungal infection of the seeds and fungal impact on morphology and physiology was investigated by FRD and LA. The molecular identification of the fungus was by EM and LDF, electron microscopy by RB. LDF performed the multiple scale data analysis, HPF the metabolite analysis by HPLC. All authors read and approved the final manuscript.
